# Identifying barriers to the availability and use of Magnesium Sulphate Injection in resource poor countries: A case study in Zambia

**DOI:** 10.1186/1472-6963-10-340

**Published:** 2010-12-16

**Authors:** Anna L Ridge, Lisa A Bero, Suzanne R Hill

**Affiliations:** 1 Medicine Access and Rational Use, Department of Essential Medicines and Pharmaceutical Policies, World Health Organization, Geneva, Switzerland; 2 Institute for Health Policy Studies, University of California at San Francisco, 1388 Sutter Street, 11th floor, San Francisco, CA 94109, USA

## Abstract

**Background:**

Pre-eclampsia and eclampsia are serious complications of pregnancy and major causes of maternal mortality and morbidity worldwide. According to systematic reviews and WHO guidelines magnesium sulphate injection (MgSO4) should be the first -line treatment for severe pre-eclampsia and eclampsia. Studies have shown that this safe and effective medicine is unavailable and underutilized in many resource poor countries. The objective of this study was to identify barriers to the availability and use of MgSO4 in the Zambian Public Health System.

**Methods:**

A 'fishbone' (Ishikawa) diagram listing probable facilitators to the availability and use of MgSO4 identified from the literature was used to develop an assessment tool. Barriers to availability and use of MgSO4 were assessed at the regulatory/government, supply, procurement, distribution, health facility and health professional levels. The assessment was completed during August 2008 using archival data, and observations at a pragmatic sample of health facilities providing obstetric services in Lusaka District, Zambia.

**Results:**

The major barrier to the availability of MgSO4 within the public health system in Zambia was lack of procurement by the Ministry of Health. Other barriers identified included a lack of demand by health professionals at the health centre level and a lack of in-service training in the use of MgSO4. Where there was demand by obstetricians, magnesium sulphate injection was being procured from the private sector by the hospital pharmacy despite not being registered and licensed for use for the treatment of severe pre-eclampsia and eclampsia by the national Pharmaceutical Regulatory Authority.

**Conclusions:**

The case study in Zambia highlights the complexities that underlie making essential medicines available and used appropriately. The fishbone diagram is a useful theoretical framework for illustrating the complexity of translating research findings into clinical practice. A better understanding of the supply system and of the pattern of demand for MgSO4 in Zambia should enable policy makers and stakeholders to develop and implement appropriate interventions to improve the availability and use of MgSO4.

## Background

Pre-eclampsia and eclampsia are serious complications of pregnancy and major causes of maternal mortality and morbidity worldwide. In 2002, there were over 4 million cases of pre-eclampsia and eclampsia globally, of which 63,000 resulted in a maternal death [1]. Estimates of case fatality rates, based mainly on hospital-based studies, show that the risk of dying from eclampsia is approximately 14 times higher in a developing country compared to a developed country [2].

Clinical evidence from systematic reviews shows that magnesium sulphate (MgSO4) should be the first -line treatment for severe pre-eclampsia and eclampsia [3-6]. Since 2003, it has been included on the World Health Organization's Model List of Essential Medicines specifically for the treatment of severe pre-eclampsia and eclampsia [7]. However, studies show that this safe and effective medicine is still unavailable and underutilized in many low and middle income countries [8,9]. The first step in designing effective interventions to increase the use of MgSO4 is to identify barriers to use and target the intervention accordingly [10].

The objective of this pilot study was to identify barriers and facilitators to the availability and use of MgSO4 in the Zambian Public Health System using a health system approach, to provide information to decision makers on the most appropriate interventions to improve the availability and use of MgSO4 for treatment of severe pre-eclampsia and eclampsia. We developed and tested a tool for rapidly assessing barriers and facilitators in the field. Zambia was chosen as the country for this case study because its maternal mortality rate is very high, 729/100 000 live births [11] and a recent logistics system assessment for reproductive health commodities indicated that MgSO4 was not widely available in health facilities in Zambia [12]. However, that assessment did not explore the underlying reasons for its unavailability and previous studies investigating the availability and use of MgSO4 injection have not included Zambia.

## Methods

### Assessment Tool Development

We developed a rapid assessment instrument, that could be used to investigate barriers and facilitators to the availability and use of MgSO4 at different levels within a health system in a resource poor setting. Identifying barriers is the first step for designing and scaling up interventions to achieve use of medicines [10]. The ability to identify and map barriers to specific areas of a health system will enable decision makers to target interventions in an efficient and cost-effective manner to improve the availability and use of MgSO4 for the treatment of severe pre-eclampsia and eclampsia. Before the rapid assessment instrument could be developed a model framework was required to identify what would need to be in place in a health system to ensure the availability and use of MgSO4.

We used a fishbone (Ishikawa or cause and effect) diagram [13] as the framework for identifying facilitators to ensure the availability and use of MgSO4. A fishbone diagram is an analysis tool that provides a systematic way of looking at effects and the causes that create or contribute to those effects. These diagrams have been used in the software and manufacturing industries, for product design, quality defect prevention, and to identify potential factors causing an overall effect. In the healthcare field, the fishbone diagram has been used most often as a continuous quality improvement tool to examine the causes of a problem within a healthcare setting. For example, Hartnell and colleagues [14] used a fishbone diagram as a an analytic approach to identify sources of medication errors in hospital. Ishikawa diagrams can be used to facilitate brainstorming exercises to identify sources of a problem or to organize information about known barriers or facilitators.

We chose the desired outcome of 'rational use of MgSO4 injection' for the treatment of severe pre-eclampsia and eclampsia, based on best available evidence and guideline recommendations [3-6,15,16] Rational use of medicines refers to the correct, proper and appropriate use of medicines [17]. To define the inputs for our fishbone diagram a literature search was undertaken to identify country studies that had specifically looked at problems of availability and/or use of magnesium sulphate for the treatment of severe pre-eclampsia and eclampsia, in low and middle income countries. The MEDLINE database was searched using the OVIDsp search engine. The search used the search term magnesium sul* combined with an individual country name and magnesium sul* combined with the term "developing countries". No time, language or study design limits were placed on the searches. The list of countries was obtained from: http://www.listofcountriesoftheworld.com/. Reference lists of relevant retrieved studies were cross checked to identify further potentially relevant studies and experts in the field were contacted. The search focussed on studies investigating barriers to the availability and use of MgSO4 and not on country data regarding incidence rates of pre-eclampsia and eclampsia. Three relevant studies were identified; two were specifically about MgSO4 [8,9]; the third was a study that described the investigation of barriers to a range of reproductive health interventions, including use of MgSO4 for eclampsia [18].

**Figure 1:**
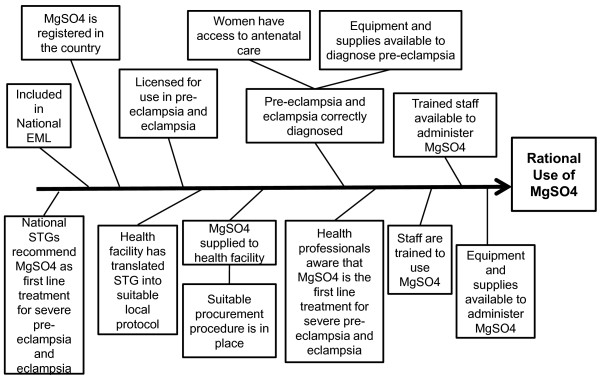
**Fishbone diagram identifying requirements for the rational use of Magnesium Sulphate in a health facility**. File contains a diagram illustrating what is required to facilitate the rational use of Magnesium Sulphate in a health facility

From the retrieved studies, we identified critical components that would need to be in place in a public health system in a resource poor setting to lead to rational use of magnesium sulphate. These included: inclusion of MgSO4 injection in the National Essential Medicines List and Standard Treatment Guidelines; registration in the country for use in the treatment of severe pre-eclampsia and eclampsia; presence of a suitable procurement procedure and distribution system; presence of a suitable local protocol developed by health facilities providing basic or emergency obstetric care; awareness of health professionals working in the facilities that MgSO4 is the first line treatment for severe pre-eclampsia and eclampsia; staff have been trained to use MgSO4; and trained staff are available to administer MgSO4 when necessary: In addition, pregnant women need to be able to access to antenatal care, equipment and supplies should be available to diagnose pre-eclampsia and equipment and supplies should be available in the health facilities to administer MgSO4. The completed fishbone diagram is shown in figure 1.

For each requirement listed in the fishbone diagram, we identified the level in the health system where the information could be gathered from review of publicly available archival materials or observations. To facilitate the rapid assessment, we focused on these data sources rather than conducting interviews or surveys. The levels identified were: government/regulatory; pharmaceutical supply system; health facilities and health professionals. Using this information we developed a rapid assessment tool (checklist) for identifying potential barriers to the availability and use of MgSO4 within the health system. Additional information for the checklist for assessing the pharmaceutical supply system came from Managing Drug Supply: the selection, procurement, distribution, and use of pharmaceuticals [19]. For the health facility section of the checklist we developed a table, based on WHO guidelines [15,16], so that a walk-through exercise could be undertaken to ascertain if the facility had sufficient quantities of MgSO4 to administer the recommended regimen for the treatment of severe pre-eclampsia and eclampsia and the necessary equipment to diagnose pre-eclampsia/eclampsia and administer and monitor the use of MgSO4. The full checklist can be found in additional file 1.

### Data collection

We wished to assess the pharmaceutical sector and supply system in Zambia in order to identify barriers to the availability of MgSO4 injection in health facilities for the management of severe pre-eclampsia and eclampsia. Barriers were assessed at the regulatory/government, pharmaceutical supply system (supply, procurement, distribution), health facility and health professional levels. An analysis of financing systems for the procurement of essential medicines in Zambia was not possible at the time of the study. The community level was not included in the assessment because the rational use of MgSO4 injection requires the patient to be in a health facility. Assessing barriers at the community level regarding access to health facilities was beyond the scope of this study.

Based on the requirements, illustrated in the fishbone diagram (figure 1), we aimed to collect data based on analysis of public sector records and documents available in the public domain and routine observations at a small pragmatic sample of health facilities providing obstetric services in Lusaka District, Zambia. Lusaka district was chosen for the pilot assessment because it has the largest population of Zambia's 72 districts and includes the national capital, Lusaka. Lusaka district contains the only Medical School in Zambia and the largest hospital in the country; the University Teaching Hospital.

### Government/Regulatory Level

At the government/regulatory level all the relevant policy documents and guidelines were obtained from the Ministry of Health via the WHO Medicines Adviser for Zambia. These documents included the most recent National Drug Policy, National Reproductive Health Policy, National Formulary, National Essential Medicines List and National Standard Treatment Guidelines. The Pharmaceutical Regulatory Authority was requested to provide information about the licensing of MgSO4 in Zambia.

### Pharmaceutical Supply System

Information about the procurement, supply and distribution of MgSO4 within the public health system of Zambia was obtained from Ministry of Health personnel responsible for the procurement of medicines for the public sector, employees at the Central Medical Store and pharmacy workers in the visited health facilities.

### Health Facilities

A walk through observational exercise was undertaken at each health facility to ascertain if the facility had the necessary supplies and equipment to: a) diagnose pre-eclampsia/eclampsia, b) administer MgSO4 injection, and c) monitor a patient receiving MgSO4 treatment of severe pre-eclampsia or eclampsia. This was to assess not only if MgSO4 was available on the day of the visit, but also if the facility had sufficient quantity of MgSO4 to give the recommended treatment regimen to one patient. Previous studies reporting the availability of medicines for emergency obstetric care (EmOC) have reported the presence or absence of medicines, but have not reported if the quantities were sufficient to provide the full course of treatment [12].

### Health Professionals

A review of available undergraduate medical and midwifery education and training materials was undertaken to determine what pre-service training health professionals were receiving for the diagnosis and management of pre-eclampsia and eclampsia.

Doctors and midwives were selected as these were the health workers most likely to be managing patients with pre-eclampsia and eclampsia and subsequently administering magnesium sulphate injection.

### Sample selection of health facilities

For this pilot assessment, a small pragmatic sample of health facilities was visited, all within Lusaka District. As there is no first level (district) or second level (provincial) referral hospitals in Lusaka District, health centres must refer complicated obstetric cases directly to the University Teaching Hospital. Three health facilities were visited: the University Teaching Hospital (UTH), that has specialist obstetric services and is the largest hospital in Zambia and two urban health centres, which were reported to be providers of basic emergency obstetric care (EmOC). A facility is described as a provider of basic EmOC when it can provide treatment for the major complications of pregnancy, including administering an anticonvulsant for the treatment of pre-eclampsia and eclampsia.

Data collection took place between 4^th ^and 14^th ^August 2008.

### Ethical approval

The research ethics committee of the WHO advised that the study protocol satisfied the requirement for audit and did not require formal approval. The Ministry of Health, Zambia gave clearance for the assessment to take place.

## Results

### Government/Regulatory Level

Magnesium sulphate injection was listed and recommended in four key policy documents - the national Essential Medicines List (2004), Zambian National Formulary (2005), the national Standard Treatment Guidelines (2004) and guidelines for pregnancy, childbirth, postpartum and newborn care (2006). In the Zambian National Formulary it is specified for use in cases of fulminant pre-eclampsia and eclampsia. The dosage regimens recommended were consistent with research evidence and international guidelines. The national Standard Treatment Guidelines (2004) and the guidelines for pregnancy, childbirth, postpartum and newborn care (2006) were not consistent in their recommendations for the use of MgSO4. The 2004 STGs gave detailed instructions for the use of MgSO4 for the management of convulsions in eclampsia, but no information for the use of MgSO4 for the prevention of convulsions in severe pre-eclampsia. The 2006 guidelines did recommend the use of MgSO4 for the management of both severe pre-eclampsia and eclampsia.

MgSO4 was not licensed in Zambia for the treatment of severe pre-eclampsia or eclampsia at the time of the assessment. In early 2008 an application was made to the Pharmaceutical Regulatory Authority (PRA) by the Ministry of Health (MoH) to have magnesium sulphate registered for supply to the public sector. At the time of the assessment, there was no record of any formal applications by private manufacturers or distributers to have MgSO4 injection licensed and the Pharmaceutical Regulatory Authority were not aware of any local companies which may be manufacturing MgSO4 injection. However, subsequent to this review the Pharmaceutical Regulatory Agency has licensed two private companies to supply MgSO4.

### Pharmaceutical logistic system

National procurement of medicines is carried out by the Ministry of Health and is based on the national Essential Medicines List. The central national medical store, (Medical Stores Limited), manages the storage and distribution of medicines for the MoH and is the main supplier of medicines to hospital pharmacies and district health offices (DHO). Central and specialized hospitals, such as the University Teaching Hospital, can get medicines from the Central Medical Store, but they are also given a grant by the MoH for the independent procurement of emergency medicines and medical supplies.

Despite MgSO4 being on the EML, it had not been procured by the MoH for several years and had been out of stock at the Central Medical Store since 2006. As a result, the district health office (DHO) for Lusaka District was also out of stock, as well as the two health centres within this district that were visited. The DHO was not using its allowance for the procurement of MgSO4, because it was reported that there was no demand from the health centres. The pharmacy at the University Teaching Hospital had MgSO4 in stock that was being procured from a local wholesaler using the grant for emergency medicines provided by the MoH. It was being procured as a result of demand for MgSO4 injection from obstetricians working at the hospital. The available stock of MgSO4 in the UTH pharmacy was found to be manufactured in Zambia.

Copies of the Zambian National Formulary, National Standard Treatment Guidelines (2004) and Essential Medicines List were not available at any of the pharmacies or stores visited. A copy of the EML 2004 was available at the Government's Central Medical Store, Medical Stores Limited.

### Health Facilities

The University Teaching Hospital was the only facility to have MgSO4 injection in stock on the labour ward on the day of the visit. MgSO4 was not available at the two health centres. However, the health centres did have the majority of the equipment and supplies necessary for the diagnosis of pre-eclampsia/eclampsia and administration of MgSO4 (see table 1). The notable exception was calcium gluconate, which according to international and national guidelines should always be available when administering MgSO4 in case of toxicity.

**Table 1:** The availability of the required equipment and supplies in the different health facilities on the day of the visit

Equipment and Supplies which are required for the use of MgSO4	Health Facility		
	
	UTH	Health Centre 1	Health Centre 2
Local treatment protocols exist for eclampsia and recommend magnesium sulphate as the first line treatment	Yes	Yes	Yes

Local treatment protocols exist for pre-eclampsia and recommend magnesium sulphate as the first line treatment	Yes	Yes	Yes

Sphygmomanometer or BP machine	Yes	Yes	Yes

Stethoscope	Yes	Yes	Yes

Dipsticks to detect protein in the urine	No	Yes	Yes

Sufficient quantity of MgSO4 to provide 24 h treatment for 1 patient	Yes	No	No

Calcium gluconate (1 g, 10 ml of 10% solution)	Yes	No	No

2% Lignocaine (1 ml ampoules)	Yes	Yes	Yes

Cannulae	Yes	Yes	Yes

Sterile syringes (10 ml or 20 ml)	Yes	Yes	Yes

Sterile needles	Yes	Yes	Yes

Sterile water or normal saline for dilution of MgSO4	Yes	Yes	Yes

Normal saline or Ringer's lactate	Yes	Yes	Yes

Drip stand	Yes	Yes	Yes

IV giving sets	Yes	Yes	Yes

Patella hammer	Yes	No	No

Urinary catheters	Yes	Yes	Yes

Urine collection bags	No	Yes	Yes

Sharps boxes for safe waste disposal	Yes	Yes	Yes

Gloves	Yes	Yes	Yes

At UTH, dipsticks for identifying proteinuria were out of stock on the day of the visit. EDL and ZNF were not available in the health facilities. The national Standard Treatment Guidelines (2004) and/or the Pregnancy, Childbirth, Postpartum and Newborn Care Guidelines (2006) were available in the health facilities visited. However, the local protocols on display in the health centres did not follow the recommended IV/IM regimen for MgSO4.

### Health Professionals

The current undergraduate educational materials for midwifery training (2004) included both theoretical and practical training elements for the management of severe pre-eclampsia and eclampsia with MgSO4. The training and educational materials for the obstretric and gynaecology rotation for medical students were not available for review. The Director of the University Teaching Hospital reported that medical students had to complete a rotation in obstetrics and gynaecology both before and after graduation and this included training in the management of pre-eclampsia/eclampsia, using MgSO4 as the first line treatment for these conditions.

All the midwives at the health facilities visited reported that they had received pre-service training in the diagnosis and management of pre-eclampsia and eclampsia. None of the midwives on duty at the time of the visit reported receiving any in-service training for the administration and monitoring of MgSO4 for the management of severe pre-eclampsia and eclampsia. It was reported that some midwives from the health centres had attended an emergency obstetric care training workshop. At the University Teaching Hospital, MgSO4 is the recommended first line treatment and the hospital has its own treatment protocol, which is consistent with research evidence and international guidelines. Checking for proteinuria was reported to be a problem due to frequent stock outs of dipsticks. Dipsticks were out of stock on the day of the visit. The University Teaching Hospital is the only referral hospital in Lusaka district for all complicated antenatal and obstetric cases. It was reported that many of the referrals from health centres are for pregnancy induced hypertension (PIH). However, on arrival at UTH the patients actually have eclampsia and require treatment with MgSO4. During the visits to the health facilities, no doctors were available to verify their pre- and post-service training in the management of pre-eclampsia/eclampsia.

See table 2 for a summary of the major barriers and facilitators identified at each level during the rapid assessment exercise

**Table 2:** Major barriers and facilitators to the availability and use of MgSO4 identified at each level

	Barriers identified	Facilitators identified	Outcome
**Government/Regulatory**	MgSO4 injection not registered in Zambia MgSO4 not licensed for use in severe pre-eclampsia or eclampsia	MgSO4 listed in Essential Medicines List and Standard Treatment Guidelines MgSO4 being manufactured in Zambia	Unregulated MgSO4 available in the market place

**Pharmaceutical supply system**	MgSO4 not being procured by MoH MgSO4 out of stock at the Central Medical Store	MgSO4 on Ministry of Health Procurement list	MgSO4 not available for delivery to health facilities

**University Teaching Hospital (UTH)**	Stock-outs of dipsticks for testing urine for proteinuria.	STG translated into suitable local protocol Health professionals aware that MgSO4 is the 1^st ^line treatment for severe pre-eclampsia/eclampsia Obstetricians requested purchase of MgSO4 Hospital pharmacy is procuring MgSO4 from local wholesaler Midwives have received training for use of MgSO4	Evidence that MgSO4 being used for treatment of eclampsia, but not severe pre-eclampsia

**Health Centre 1** **Health Centre 2** **(findings at the two health centres were the same)**	MgSO4 out of stock Midwife on duty had never administered MgSO4 Local protocol did not follow evidence-based guidelines for use of MgSO4	Midwife on duty aware that MgSO4 is the first line treatment for severe pre-eclampsia and eclampsia Evidence-based guidelines available Equipment and supplies available to diagnose pre-eclampsia Equipment and supplies available to administer MgSO4	No evidence of use of MgSO4. Cases of eclampsia treated with diazepam and referred to UTH

## Discussion

Our study is the first study we are aware of to try to identify barriers to the availability and use of MgSO4 using a health system approach. We found that the current situation in Zambia shows that research knowledge regarding magnesium sulphate for the treatment of severe eclampsia and eclampsia has been translated into policy. However, implementation of policy into practice was found to be limited at the lower levels of care. Numerous barriers to the availability and use of MgSO4 were identified within the health system, including a lack of stock. There was a lack of dissemination of national standard treatment guidelines in health facilities and pharmacies and a general lack of in-service training for both midwives and pharmacists regarding the correct use of MgSO4. The demand for MgSO4 at the health centre level was reported to be low. However, as this assessment did not include an audit of clinical data it is not clear if this was because there were in fact few cases or that cases were being misdiagnosed. There can be no demand if there is failure to diagnose the condition. It should be noted that the health centres visited during the study period did have the necessary equipment and medical supplies to diagnose pre-eclampsia and eclampsia. At the health centre level, due to the low demand from the midwives, MgSO4 was not considered to be a priority medicine by the pharmacists in charge of procurement. As a result they were not putting pressure on the Central Medical Store to make it available.

While pre-eclampsia and eclampsia are clearly important causes of maternal mortality globally, the absolute frequency of cases presenting to an individual facility may be low. Although we did not assess clinical records, the anecdotes from staff suggested that cases of pre-eclampsia were infrequent at small hospitals. If this is correct, then lack of recent knowledge and experience would be another potential barrier to effective administration of MgSO4 and would highlight the need for frequent refresher courses and other educational reminders, to ensure appropriate diagnosis and treatment.

Poor availability of magnesium sulphate may also reflect limitations of procurement systems. Magnesium sulphate was not supplied to the lower levels of care because it was out of stock at the Central Medical Store. The demand for MgSO4 at the health centre level was apparently low, but it is not clear if the problem of availability was due to lack of demand leading to a lack of supply or vice versa.

During the development of the rapid assessment tool it was suggested that the lack of licensing of MgSO4 injection was a key potential barrier to the availability and use of MgSO4 and this may have contributed to it not being available at the district facilities. The University Hospital Pharmacy, following demand from obstetricians, was able to procure locally manufactured magnesium sulphate, but the lack of registration clearly raises uncertainty about the quality of the product. As an injectable medicine, the manufacture of magnesium suphate needs to be closely monitored to ensure that the ampoules are not contaminated during the manufacturing process.

In the walk through exercise at health facilities, apart from the quantity of MgSO4 required for the treatment of one patient for 24 hours, only the presence or absence of the other essential supplies and equipment required for the diagnosis, administration and monitoring of MgSO4 was evaluated, and not the quality or quantity of them. Clearly, the total stock and quality of supplies and equipment could have a bearing on the actual delivery of care and rational use of MgSO4 for the treatment of severe pre-eclampsia and eclampsia.

The main limitation of this case study is that it involved a small number of facilities in one district in Zambia, in the capital city and thus the findings may not reflect the current situation in other districts/provinces of Zambia. However, it is likely that MgSO4 availability in the capital city and surrounding district represents the most optimistic picture of the supply and use situation within Zambia. Follow-up studies should include public and private sector health facilities and pharmacies in other districts, including rural areas. Other limitations of our study include the lack of financial data regarding the cost of MgSO4 and its affordability, and the lack of information regarding the availability of MgSO4 injection in the private sector.

Ideally, qualitative methods could have been used to assess the knowledge and experience of staff at health facilities in relation to use of magnesium sulphate. However, our intention was to develop a rapid assessment instrument that might be applied in a variety of settings. Therefore our data sources needed to be publicly available archival materials and observations. Future assessments could be expanded to incorporate a qualitative method to uncover potential barriers to the use of MgSO4 injection by doctors and midwives in clinical practice. The role of community members, community health workers and traditional birth attendants in recognizing risk factors for pre-eclampsia and development of eclampsia was beyond the scope of this study. However, it should be noted that educational interventions to increase awareness of the risk factors for the development of severe pre-eclampsia/eclampsia at this level may have an impact on the number of referrals to health facilities and consequently on demand for MgSO4.

Since undertaking our study we have become aware of other frameworks that have been used to analyse access to health technologies in resource poor settings [20]. There is still much to learn in this complex area and awareness of different approaches will enable the ultimate assessment instrument to be developed. Although our instrument does have limitations, it gathers useful information on barriers in an efficient way without disruption of health care provision.

The successful translation of research evidence into clinical practice is a complex process. Many studies [9,21,22] have concentrated on mechanisms for translating research evidence into policy. What is suggested by this assessment is that although the policy makers - as reflected in essential medicines list and treatment guidelines - have been persuaded by the evidence, the gap in this case is the translation from policy into action plans. To close this gap requires an integrated approach throughout the pharmaceutical sector and pathways for clinical care that simultaneously responds to all of the factors identified in the fishbone diagram. Such a strategy would include acting on the recommendations listed in Table 3. The challenge is developing methods to implement such a strategy successfully in fragile health systems and resource limited settings. In order to determine if our recommendations are sufficient and appropriate for turning policy into practice further operational research is required. Our recommendations need to be implemented and evaluated using a rigorous methodology.

**Table 3:** Recommendations for overcoming the barriers identified

1. National treatment guidelines need to be up-dated to include MgSO4 for the treatment of severe pre-eclampsia, and the National Essential Medicine List and National Formulary need to be disseminated to all health facilities providing antenatal and delivery care, and to pharmacists working in the government stores and health facility pharmacies.
2. Magnesium sulphate needs to be registered with the PRA in order to ensure its safety, efficacy and quality.
3. Magnesium sulphate needs to be stocked at the Central Medical Store so that it can be made available to district hospitals and health centre
4. The market demand for MgSO4 needs to be determined, probably including a prospective study of the incidence of eclampsia and pre eclampsia.
5. In-service training for midwives and pharmacists needs to be co-ordinated with the availability of magnesium sulphate at the Central Medical Store, to avoid long periods between the training and using the product, and reinforcing the need for supply.

## Conclusions

The study highlights the complicated issue of ensuring that essential medicines are available and used appropriately. The fishbone diagram is a good way to illustrate the complexity of translating research findings into clinical practice. Access to a medicine is a complex construct because medicines not only have to be available within the health system, they have to be prescribed and in some cases, such as MgSO4, also administered by a health professional, before patients can benefit from their effects and health outcomes can be improved. Much work remains to be done at the level of individual countries to understand these factors better. There is a need to move beyond simply determining whether a medicine is available in the Central Medical Store or health-care facility towards considering prescribing practices, the relevance and appropriateness of standard treatment guidelines and factors that facilitate appropriate use by health professionals.

This study shows that it is feasible to undertake a system based approach for assessing availability of key essential medicines. Ideally, it should be complemented by independent interviews or focus groups to determine the knowledge and understanding of the use of the medicines concerned. However, even without that information, it is possible to identify a number of barriers to the use of magnesium sulphate that can be addressed, such as the licensing of an appropriate product, supply and dissemination of relevant treatment guidelines, and educational reminders. Any intervention to promote use of magnesium sulphate as a tool for reducing maternal mortality will need to take these factors into account.

## Competing interests

The authors declare that they have no competing interests.

## Authors' contributions

ALR devised the study protocol, developed the checklist, collected the data and drafted the manuscript.

LAB advised on the framework for assessment and edited drafts of the manuscript.

SRH advised on the study protocol and edited drafts of the manuscript.

All authors read and approved the final manuscript.

## Pre-publication history

The pre-publication history for this paper can be accessed here:

http://www.biomedcentral.com/1472-6963/10/340/prepub

## Supplementary Material

Additional file 1 **Checklist for identifying barriers to the availability and use of Magnesium Sulphate Injection**. File contains the rapid assessment tool used to collect information for the study. Click here for file
