# Supplementary material for: Identifying barriers to the availability and use of Magnesium Sulphate Injection in resource poor countries: A case study in Zambia
Source: BMC Health Serv Res. 2010 Dec 16;10:340. doi: 10.1186/1472-6963-10-340 (PMC3018452; doi:10.1186/1472-6963-10-340)
Supplement: Additional file 1 — Checklist for identifying barriers to the availability and use of Magnesium Sulphate Injection. File contains the rapid assessment tool used to collect information for the study. [file 1472-6963-10-340-S1.DOC]

**Government/Regulatory level**

| **National Policies supporting the availability and use of MgSO4 injection** | **Yes** | **No** | **Date of last revision/date MgSO4 added** |
| --- | --- | --- | --- |
| The current National Drug Policy supports the essential medicine concept |  |  |  |
| The current National Reproductive Health Policy supports access to ANC and provision of Emergency Obstetric Care |  |  |  |
| The current National Formulary lists MgSO4 for treatment of pre-eclampsia and eclampsia |  |  |  |
| MgSO4 is listed in the current National Essential Medicines List |  |  |  |
| National clinical guidelines/standard treatment guidelines (STGs) exist for the treatment of pre-eclampsia and eclampsia |  |  |  |
| In the current STGs MgSO4 is indicated for the treatment of eclampsia |  |  |  |
| In the current STGs MgSO4 is indicated for the treatment of pre-eclampsia |  |  |  |
| MgSO4 is currently registered in the country |  |  |  |

Other supportive information:

What level of health facility is MgSO4 approved for?

What other drugs are licensed and indicated for pre-eclampsia and eclampsia?

Are these drugs included in the NEDL and national clinical guidelines?

(Obtain copies of all the policy documents and guidelines reviewed)

**Pharmaceutical logistic system**

Provide a brief description of how procurement of medicines is done in the country

Who plans for selection?

Who plans for procurement?

Describe who is involved in the procurement process, i.e. government, donors, UN organizations, NGOs

Where do the estimates for quantity of magnesium sulphate to be procured come from?

Provide a brief description of how medicines are distributed to health facilities in the country

| **Requirements for availability of MgSO4 injection** | **Yes** | **No** | **Don't know** |
| --- | --- | --- | --- |
| There is a functional procurement system in place |  |  |  |
| MgSO4 is being procured by the MoH |  |  |  |
| There is a functional distribution system in place |  |  |  |
| Quantities of magnesium sulphate to be procured are based on actual consumption |  |  |  |
| Magnesium sulphate is in stock at the central medical store |  |  |  |
| Stock outs of magnesium sulphate do not occur at the central medical store |  |  |  |

**Checklist for Individual Health Facility Review**

Name of health facility: ______________________________________________

Type/ level of health facility: __________________________________________

| **Current members of staff** | **Total Number** | **Number per shift** |
| --- | --- | --- |
| Obstetricians |  |  |
| General Doctors |  |  |
| Midwives |  |  |
| Nurses |  |  |
| Medical Assistants |  |  |
| Pharmacists |  |  |
| Pharmacy Technologists |  |  |

**Pharmacy at health facility**

Review the availability of magnesium sulphate injection at the health facility’s pharmacy.

| **Information required** | **Details** |
| --- | --- |
| Availability of stock management record at facility for MgSO4 injection |  |
| Ordering system for MgSO4 injection at facility |  |
| MgSO4 injection in stock on day of visit |  |
| Current stock level |  |
| Source of MgSO4 injection |  |
| If not in stock, date last in stock |  |
| Availability of NF, EDL or STGs |  |
| Pharmacist/Pharmacy Technologist has received in-service training regarding use of MgSO4 injection for treatment of pre-eclampsia/eclampsia |  |

Review and document stock card information for magnesium sulphate injection

If information is available, record the dates and duration of any stock-outs for MgSO4 injection:

Calculate the total number of stock-out days in a 12 month period counting starting from day of the visit

Calculate the total consumption of MgSO4 in a 12 month period counting starting from day of the visit

Calculate the Average Monthly Consumption (AMC) adjusted for stock-outs:

AMC = Total consumption ÷ (Total no. of days in period) - (stock-out days)

Average no. of days in month

Calculate the number of months in stock:

Number of months in stock = Current stock level ÷ AMC

**Labour Ward at health facility**

Carry out a walk through exercise on the labour ward to review the availability of the equipment and supplies required to facilitate the use of magnesium sulphate injection for the treatment of severe pre-eclampsia and eclampsia

| **Requirements for use of MgSO4 injection** | **Yes** | **No** | **Don't know** |
| --- | --- | --- | --- |
| Current National formulary available in health facility |  |  |  |
| Current STGs available in health facility |  |  |  |
| Current National Essential Medicines List available in health facility |  |  |  |
| Local treatment protocols exist for eclampsia and recommend magnesium sulphate as the first line treatment |  |  |  |
| Local treatment protocols exist for pre-eclampsia and recommend magnesium sulphate as the first line treatment |  |  |  |
| Sphygmomanometer or BP machine |  |  |  |
| Stethoscope |  |  |  |
| Uristix to detect protein in the urine |  |  |  |
| Sufficient quantity of MgSO4 to provide 24h treatment |  |  |  |
| Calcium gluconate (1g, 10ml of 10% solution) |  |  |  |
| 2% Lignocaine (1ml ampoules) |  |  |  |
| Cannulae |  |  |  |
| Tape to secure cannula |  |  |  |
| Sterile syringes (10ml or 20ml) |  |  |  |
| Sterile needles |  |  |  |
| Sterile water or normal saline for dilution of MgSO4 |  |  |  |
| Normal saline or Ringer's lactate |  |  |  |
| Drip stand |  |  |  |
| IV giving sets |  |  |  |
| Patella hammer |  |  |  |
| Urinary catheters |  |  |  |
| Urine collection bags |  |  |  |
| Sharps boxes for safe waste disposal |  |  |  |
| Gloves |  |  |  |

**Quantity of magnesium sulphate required for 24 hrs of treatment:**

A 10 ml ampoule of 50% solution contains 5g of MgSO4.

A 2ml ampoule of 50% solution contains 1g of MgSO4.

If using a 10ml ampoule of 50% solution:

- IV/IM regimen requires 44g or 10 ampoules
- IV regimen requires 28g or 6 ampoules

If using a 2ml ampoule of 50% solution:

- IV/IM regimen 44g or 44 ampoules
- IV regimen 28g or 28 ampoules

Other supportive information:

- Is the vial easy to open?
- Do the STG/local protocols explain how to prepare MgSO4 for administration?
- Are the guidelines clearly written and easy to follow?
- What other anticonvulsants are available at the health facility for the treatment of pre-eclampsia and eclampsia?

**Health Professionals**

Carry out a review of the medical, pharmacy, midwifery and nursing curricula to determine what pre-service training health professionals are receiving for the diagnosis and management of pre-eclampsia and eclampsia

| **Curriculum contains information regarding:** | **Diagnosis of pre-eclampsia** | | | **Diagnosis of eclampsia** | | | **MgSO4 is the first-line treatment** | | | **Date of last revision** |
| --- | --- | --- | --- | --- | --- | --- | --- | --- | --- | --- |
|  | **Yes** | **No** | **Don’t know** | **Yes** | **No** | **Don’t know** | **Yes** | **No** | **Don’t know** |  |
| Obstetricians |  |  |  |  |  |  |  |  |  |  |
| General Doctors |  |  |  |  |  |  |  |  |  |  |
| Midwives |  |  |  |  |  |  |  |  |  |  |
| Nurses |  |  |  |  |  |  |  |  |  |  |
| Medical Assistants |  |  |  |  |  |  |  |  |  |  |
| Pharmacists |  |  |  |  |  |  |  |  |  |  |
| Pharmacy Technologists |  |  |  |  |  |  |  |  |  |  |

| **Requirements for use of MgSO4 injection** | **Yes** | **No** | **Don't know** |
| --- | --- | --- | --- |
| Staff able to diagnose pre-eclampsia |  |  |  |
| Staff able to diagnose eclampsia |  |  |  |
| Staff have access to educational material (medical journals, internet and library resources) |  |  |  |
| Staff aware of evidence and current best practice for the treatment of eclampsia |  |  |  |
| Staff aware of evidence and current best practice for the treatment of pre-eclampsia |  |  |  |
| Staff believe evidence is applicable to their setting |  |  |  |
| Staff trained in the administration and monitoring of MgS04 |  |  |  |

Other supportive information:

- Which health professionals are allowed to prescribe magnesium sulphate?
- Which health professionals are allowed to administer magnesium sulphate?

STG should be used for in-service training, supervision and medical audit:

- Have health professionals received any specific in-service training in the use of magnesium sulphate injection for the treatment of pre-eclampsia and eclampsia?
- If yes:
- When did this training take place?
- What did it involve?

To investigate how actual practice relates to the national and local guidelines it is necessary to review the labour ward registry for cases of pre-eclampsia and eclampsia. The medical records of these cases then need to be reviewed to determine:

- What was prescribed to treat the pre-eclampsia or eclampsia?
- If magnesium sulphate was not prescribed, what was used instead?
- Was the treatment in line with the local health facility treatment protocol and the national guidelines?

| **Estimated workload of facility** | **Number in last year** | **Comment if information is not available** |
| --- | --- | --- |
| Pregnant women attending for ANC at facility |  |  |
| Women who deliver at facility |  |  |
| Number of recorded cases of pre-eclampsia in last year |  |  |
| Number of recorded cases of eclampsia in last year |  |  |

**Pregnant women:**

If women do not have access to ANC, complications of pregnancy will not be diagnosed. They will also not receive information and advice about the complications of pregnancy. The assumption is that information and education regarding the warning signs and symptoms of pre-eclampsia and eclampsia will facilitate pregnant women to seek medical advice and treatment early.

Other supportive information:

- What is the percentage of pregnant women that attend health facilities for antenatal care?
- What percentage have their BP measured and urine checked for protein?
- Number of visits and type of investigations carried out.

Possible source of information:

- Most up-to-date demographic health survey for the country
